# Transformer Winding Deformation Detection Based on BOTDR and ROTDR

**DOI:** 10.3390/s20072062

**Published:** 2020-04-07

**Authors:** Shuguo Gao, Yunpeng Liu, Huan Li, Lu Sun, Hongliang Liu, Qun Rao, Xiaozhou Fan

**Affiliations:** 1 State Grid Hebei Electric Power Research Institute, Shijiazhuang 050021, China; gaoshuguol@163.com (S.G.); 2 State Key Laboratory of Alternate Electrical power system with Renewable Energy Sources, North China Electric Power University, Baoding 071003, China; 3 Hebei Provincial Key Laboratory of Power Transmission Equipment Security Defence, North China Electric Power University, Baoding 071003, China

**Keywords:** transformer, winding deformation, distributed fiber sensing, Brillouin scattering, Raman scattering

## Abstract

In order to realize distributed measurement of transformer winding temperature and deformation, a transformer winding modification scheme with a built-in distributed optical fiber was designed. By laying a single-mode fiber and a multi-mode fiber on the transformer winding, the Brillouin optical time domain reflection technique (BOTDR) and the Raman optical time domain reflection technique (ROTDR) are used to measure the strain and temperature of the winding to complete the more accurate winding deformation detection. The accuracy of strain and temperature sensing of this scheme was verified by simulation. Then, according to the scheme, a winding model was actually wound, and the deformation and temperature rise tests were carried out. The test results show that this scheme can not only realize the deformation detection and positioning of the winding, but can also realize the measurement of the winding temperature; the temperature measurement accuracy reached ±0.5 °C, the strain measurement accuracy was 200 με, and spatial resolution was up to 5 m. In this experiment, the deformation location with the precision of 2 turns was realized on the experimental winding.

## 1. Introduction

The transformer is an important part of the power system. Its safe and stable operation is an important guarantee for the stability of the power system. According to statistics, the transformer winding deformation is the cause of more than 50% of transformer failures [1], so monitoring the transformer winding deformation is crucial.

Conventional winding deformation detection includes the short-circuit impedance method, the low-voltage pulse method [2], the frequency response method [3,4,5], the vibration method [6], ultrasonic methods [7,8], etc. Most of these methods have been widely used in power systems and have formed industry standards. However, there are still problems such as difficulty in fault location, low detection accuracy, and vulnerability to external interference.

Distributed optical fiber sensing is a new sensing method that has emerged in recent years. It relies on optical fiber as a transmission and sensing medium to sense the information on the entire length of the fiber and thereby achieves distributed measurement. The fiber component is silica, which is extremely stable in nature and has been widely used in bridges [9], buildings [10], railways and other electric equipment [11]. The excellent insulation properties of the fiber, the extremely small size (the normal tight casing diameter is only 0.9 mm) and the good electromagnetic compatibility make it a bright prospect in the power industry [12].

The Brillouin optical time domain reflectometry (BOTDR) technique relies on the frequency shift of the backward Brillouin scattered light in the fiber, and can detect the strain and temperature information along the fiber. Liu et al. [13] describe the use of Brillouin scattering combined with optical fibers on the windings to diagnose the transformer winding deformation. However, due to the characteristics of Brillouin frequency shift (BFS), that is sensitive to both strain and temperature, the effect of temperature on the BFS has not been eliminated in the literature, which makes it extremely limited in practical applications. In order to achieve the decoupling of temperature and strain, there are the following methods: [11,14]:

(1) Two fibers are laid side by side, one of which remains slack as a reference fiber and only senses temperature change. Use this temperature information to compensate the parallelly laid sensing fibers.

(2) Using the Brillouin scattering peaks’ height sensitivity to temperature and strain, obtain the simultaneous peak height and BFS to solve.

(3) Determine the multi-peak Brillouin scattering spectrum using a special optical fiber such as a large effective area non-zero dispersion shifted fiber (LEAF). The strain coefficients corresponding to each peak are not equal, and the strain information can be obtained by merging and solving the equation by frequency shift of multiple peaks [15].

(4) Temperature measurement is performed using a Raman time domain reflection technique (ROTDR), and temperature compensation is performed on the BFS according to the measured temperature.

Liu et al. [16] designed a scheme for grooved winding with a sensing fiber in it, then using ROTDR and BOTDR to detect the winding deformation. However, the design of the slotted wire has greatly changed the structure of the wire, which has reduced its ampacity and short-circuit resisting ability, and it is difficult to extract the fiber in the actual production process of the transformer. Liu et al. [17] designed a model that uses epoxy resin adhesive to fix fibers to the winding to achieve distributed temperature sensing of windings, but epoxy resin adhesive has strong fluidity; it is neither convenient nor efficient to use epoxy resin to fix fibers.

On the basis of the previous research, a new type of oil-immersed transformer winding with built-in fiber is developed in this paper. The fiber is wrapped with insulating paper. This process is more efficient than other optical fiber arrangements. Later, combined ROTDR and BOTDR technologies were used to detect the winding deformation. Experiments, using ROTDR temperature measurement, can achieve good results for temperature compensation for BFS, can accurately decouple the strain information, and locate the winding deformation position. At the same time, accurate temperature information of the windings can also be obtained. This allows the technology to be used on an operating transformer, and significantly improves the intelligence of transformer operation and maintenance.

## 2. Detection Principle

### 2.1. Optical Time Domain Reflectometry (OTDR)

Optical time domain reflection technology [18] is the basis of distributed fiber sensing positioning. When a laser beam is incident on the fiber, the photon will interact with the fiber medium to cause backscattering while the beam propagates forward along the fiber. By measuring the time *t* of the return of the scattered light, the distance from the end of the fiber *l* where the scattering event occurs can be obtained by Equation (1).

(1) $$t=\frac{2nl}{c},$$

where *n* is the effective refractive index of the fiber core and *c* is the speed of light in a vacuum.

### 2.2. Brillouin Optical Time Domain Reflectometry (BOTDR)

Brillouin scattering in the fiber is a nonlinear light scattering phenomenon in which the incident photon interacts with the elastic phonon in the fiber to cause the scattered light to shift relative to the incident light. The frequency shift is linear with temperature and strain. As shown in Equation (2)

(2) $$\nu_{B}=\nu_{B0}+C_{T}\Delta T+C_{\varepsilon }\Delta \varepsilon ,\;$$

where *ν_B_*s the Brillouin frequency (BF), *ν_Β_*_0_ is the BF initial value, and *C_T_* and *C*ε are the temperature and strain coefficients, respectively. *ΔΤ* and *Δε*are the change of temperature and strain relative to the initial conditions.

### 2.3. Raman Optical Time Domain Reflectometry (ROTDR)

Raman scattering is an inelastic scattering caused by the interaction of photons and crystal molecules with vibration, rotation, and the like. The scattering line consists of a lower frequency Stokes light and a higher frequency anti-Stokes light distributed across the Rayleigh scattering line. The ratio of the intensity of the two scattered lights is only related to temperature. As shown in Equation (3), the temperature of the fiber can be solved by derivation [19]:

(3) $$\frac{I_{as}}{I_{s}}={\left(\frac{\nu_{a}}{\nu_{s}}\right)}^{4}\exp\left[-\left(\frac{h\Delta \nu }{kT}\right)\right],$$

where *I_as_* and *I_s_* are the intensity of anti-Stokes and the intensity of Stokes, respectively, *ν*_as_ and *ν*_s_ are the frequencies of Stokes scattered photons and anti-Stokes scattered photons, respectively, *ν* is the Raman energy level difference, *h* is Planck‘s constant, *k* is the Boltzmann constant and *T* is the absolute temperature.

### 2.4. Temperature Compensation

It can be seen from Equation (2) that the BF is sensitive to temperature and strain, and the internal temperature distribution of the transformer is not uniform, so if only the Brillouin frequency shift is measured, accurate winding deformation detection cannot be achieved [19,20]. Since the ROTDR technology is not affected by the strain along the fiber, this paper uses the ROTDR technology to detect the winding temperature to compensate for the Brillouin frequency shift caused by the temperature, so as to obtain the accurate strain of the winding. In this paper, two fibers are laid side by side, one single-mode fiber (SMF) for BOTDR measurement and the other multi-mode fiber (MMF) for ROTDR measurement. During the laying process, the length and routing of the two fibers need to be kept consistent to ensure that the data points at the same distance of the two measurement curves correspond to the same position of the winding.

For the point *i* at the distance *x_i_* from the end of the fiber, there is Equation (4):

(4) $$\{\begin{array}{c} T_{i}=T_{Ri}\\ \nu_{Bi}=\nu_{B0i}+C_{T}\Delta T_{i}+C_{\varepsilon }\Delta \varepsilon_{i} \end{array}.$$

In the equation, the subscript i represents the value of the corresponding physical quantity at *x_i_*, and *T_Ri_* represents the temperature value measured by Raman scattering.

From Equation (4), the temperature compensation formula can be solved jointly:

(5) $$\Delta \varepsilon_{i}=\frac{\nu_{Bi}-\nu_{B0i}-C_{T}\cdot \Delta T_{i}}{C_{\varepsilon }}$$

## 3. Experiment Design

### 3.1. Selection of Fiber

**Figure 1:**
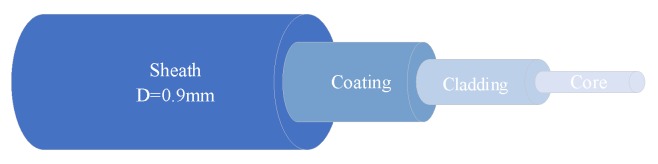
Structure of optical fiber.

As shown in Figure 1, the optical fibers are composed of a core, a cladding, a coating layer and a sheath from the inside to the outside.

The fiber core material is silica, its chemical properties are extremely stable, and its maximum withstand temperature is much higher than the highest temperature that may occur inside the transformer.

The coating material on the outer side of the core is generally polyimide, which is wrapped by the outer sheath and generally does not have direct contact with the transformer oil; its thermal stability also ensures continuous operation in the transformer environment.

The fiber sheath material is generally an organic polymer, and its stability is crucial for the safe operation of the transformer and the reliability of the fiber sensing. Based on our previous research [21,22,23], ethylene tetrafluoroethylene, ethylene-four fluoroethylene copolymer (ETFE) sheath, which can withstand temperatures up to 150 °C, is chemically stable, has no obvious effect on the overall insulation performance of oil, and is suitable for use in oil-immersed transformers.

Based on the above analysis, we selected YOFC 50/125 (OM3-300) high temperature resistant multi-mode fiber (MMF) and YOFC 9/125 (G657-A1) single mode fiber (SMF) as the sensing fiber. The minimum bending radius is 50 mm.

### 3.2. Calibration of Fiber Strain and Temperature Coefficient

**Figure 2:**
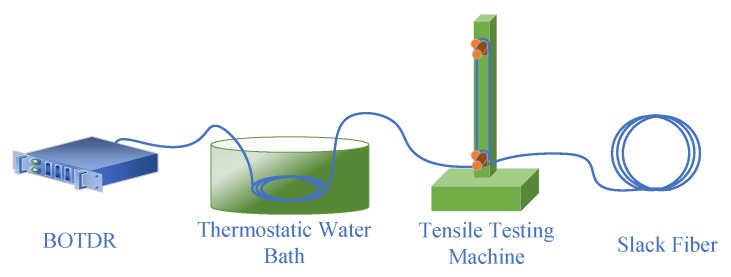
Fiber calibration device diagram.

According to Equation (5), in order to accurately decouple the strain information from the BFS measured by the BOTDR, the temperature coefficient *C_T_* and strain coefficient *C_ε_* of the single-mode ETFE fiber need to be calibrated. The calibration is performed using the device shown in Figure 2 below.

**Figure 3:**
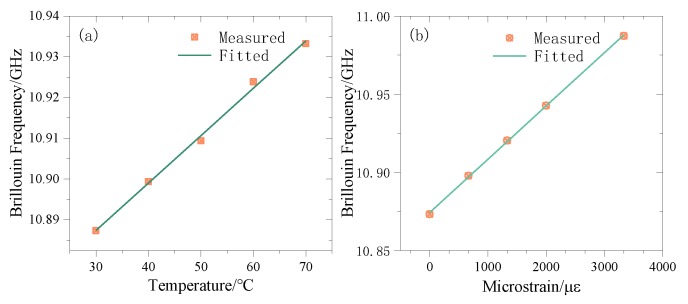
Schematic diagram of the fiber temperature and strain coefficient fitting. (**a**) BFS-temperature fitting curve (**b**) BFS-strain fitting curve.

The temperature calibration part is placed in a CS601 thermostatic water bath using about 100 m slack SMF. The temperature of the water bath is set from 30 to 70 °C in 10 °C steps. The strain calibration part uses a JF-9003 tensile tester, using silicone rubber to fix 6 turns of a total of 9 m optical fiber, stretching them 5 times in 0.5 mm steps. Record the BF in the heated and stretched area and fit the corresponding variables respectively, as shown in Figure 3.

The calibration shows that the temperature coefficient of the SMF is *C_T_* = 1.16 MHz/°C, R^2^ = 0.9964, the strain coefficient is C*_ε_*=0.033 8MHz/με, R^2^=0.9996.

### 3.3. Transformer Winding Design With Built-in Fiber

**Figure 4:**
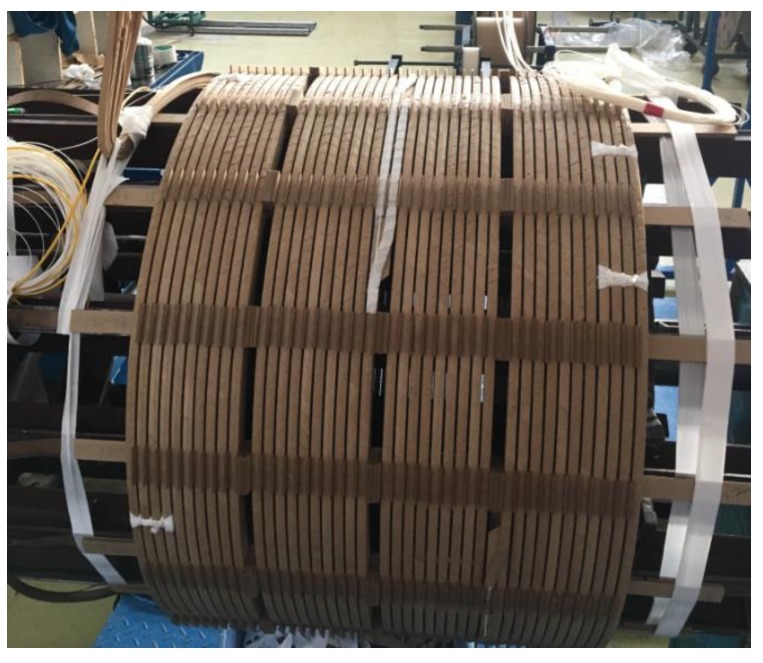
Winding with build-in fiber.

In order to monitor the full length of the winding, a winding model was made based on the size of a low-voltage winding of a 31.5 MVA 110 kV transformer. Winding with 8 rectangular copper wires, using helical winding, a total of 40 turns, and with a diameter of 700 mm, a winding model is finally produced which shown in Figure 4.

**Figure 5:**
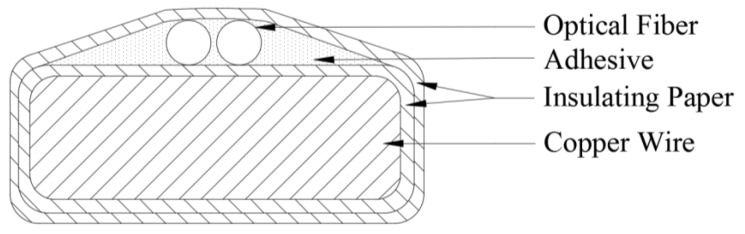
Built-in fiber winding cross-section.

When the transformer winding is deformed by a short-circuit impact, it will generally appear convex or concave in the same strip interval [1,24]. If the fiber is laid on the inside of the wire, it is easily squeezed, resulting in excessive transmission loss or even fiber fracture. The inner-fiber-wire plan was not adopted due to the processing difficulty and low efficiency. Therefore, we designed a process of laying two fibers side by side on the outside wire of each turn of the winding, wrapped with insulating paper and fixed with latex, so that there is no relative sliding between the fiber and the winding. The winding cross-section is shown in Figure 5. At the wire transposition, the fiber is also indexed with the wire to ensure that the fiber is always attached to the outermost side of each wire turn [13,17,25].

In order to ensure that the signal is extracted and the winding part of the signal is not affected by the fiber end reflection, both fibers left 20 m at the beginning and the end of the winding.

### 3.4. Research on Winding Sensing Accuracy

In order to ensure that this scheme can effectively sense the strain occurring on the winding, and also ensure the accuracy of temperature compensation, i.e., there should not be significant difference between the temperature of the two optical fibers and the temperature of the winding during the operation of the transformer.

First, the strain transfer characteristics of the winding model were analyzed. A small section of winding between two spacers was selected for research. The following simulation model was established using COMSOL Multiphysics. The simulation material parameters are shown in Table 1 below. Uniform stress is applied to the inner surface of the winding to simulate the electromagnetic force experienced during a short circuit. The stress varies from −600 to 600 KPa with a step size of 200 KPa.

**Figure 6:**
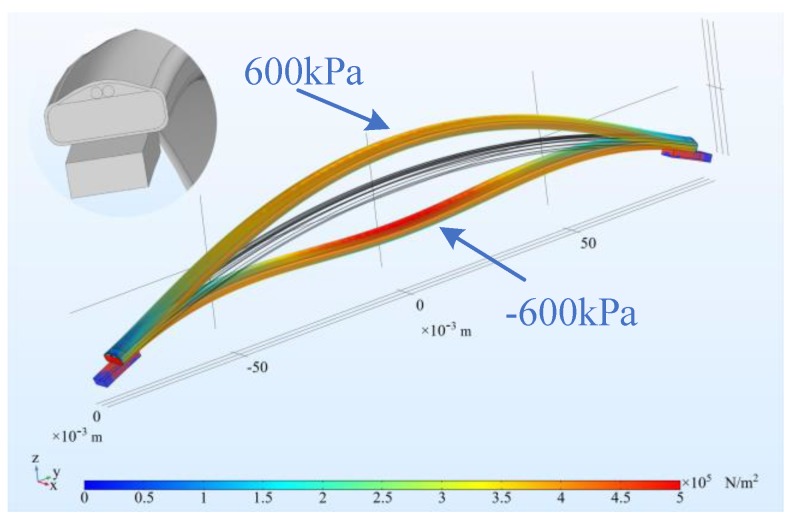
Strain transfer model and the deformation result.

**Figure 7:**
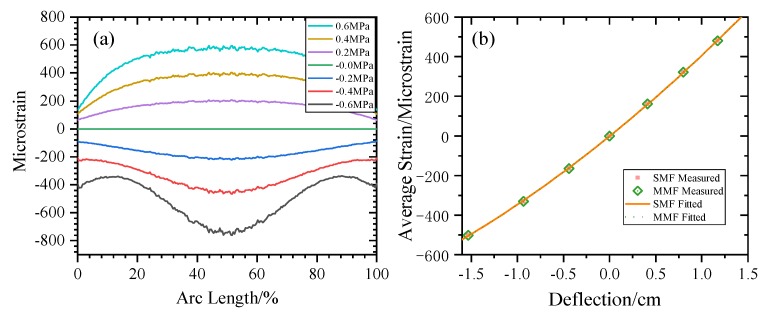
Relationship between winding deformation and strain. (**a**) The distribution of strain varies with the magnitude of stress. (**b**) the relationship between average stress and deflection.

The simulation model cross section and results are shown in Figure 6 and Figure 7. It can be seen in Figure 7a that when the winding has a concave deformation, the fiber strain is negative, that is, it is compressed, and its BFS will be negative; when the winding has a convex deformation, the fiber strain is positive, that is, it will be stretched, and the BFS will be positive. Figure 7b shows that the deflection of the winding deformation has a quadratic function relationship with the average strain on fiber, i.e., the BFS, R^2^ = 0.9999, which makes it possible to calculate the degree of winding deformation from the BFS.

**Figure 8:**
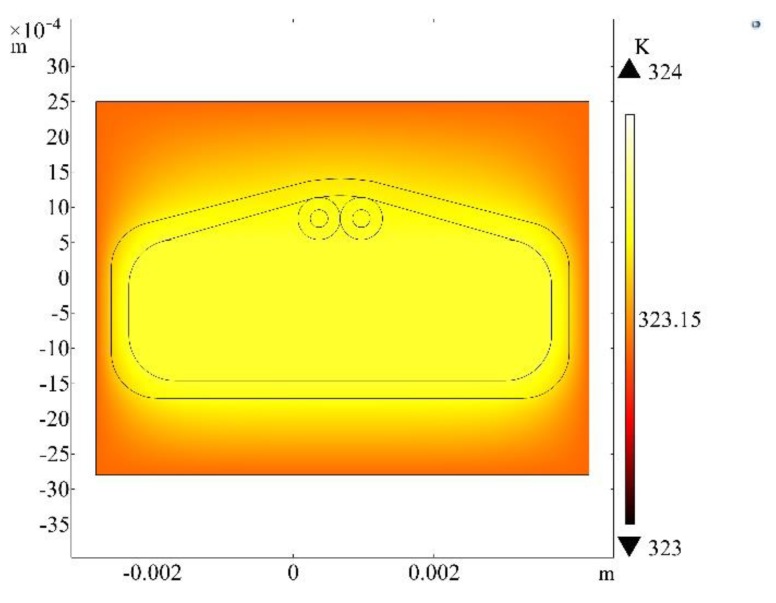
Winding temperature field distribution.

In order to verify the accuracy of temperature compensation, COMSOL Multiphysics was used to analyze a small neighborhood around the fiber composite conductor, and the following temperature field simulation model was established. The material parameters are shown in Table 1. External oil temperature is set to 50 °C, and the heating power of the wire is 0.05 W. The model boundaries are open boundaries, which simulate a single-turn winding in an operating transformer. The results are shown in Figure 8 below. The results show that the temperature difference between the winding and the optical fiber and the two fibers are within 1 °C, which proves that the dual-fiber joint measurement method can effectively achieve the BFS in actual transformer operation temperature compensation.

## 4. Experimental Research

### 4.1. Determination of the Original Curve

After the winding is produced, it is allowed to stand for 48 h to cure the latex, and at the same time the residual stress during winding can be released. Because the internal space of the experimental hall is large enough, it can be considered that the temperature of the hall changes very slowly, and the windings are in a thermal equilibrium state, that is, the winding parts and the fiber temperature are the same as the room temperature. This state is used as the original state of the winding BOTDR and ROTDR tests.

The BOTDR test uses a distributed optical fiber stress detection system produced by Weihai Beiyang Optoelectronic Info-Tech Co., Ltd. The parameters are set as Table 2 shows:

Under these parameter settings, the test time is 13 min.

**Figure 9:**
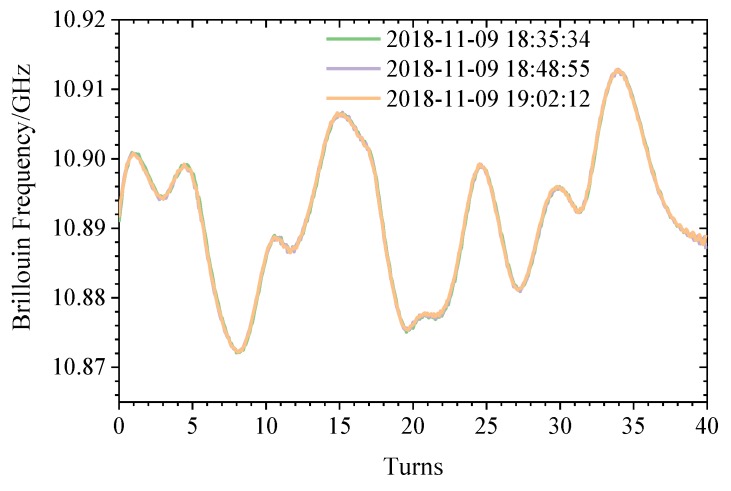
Winding original frequency shift.

**Figure 10:**
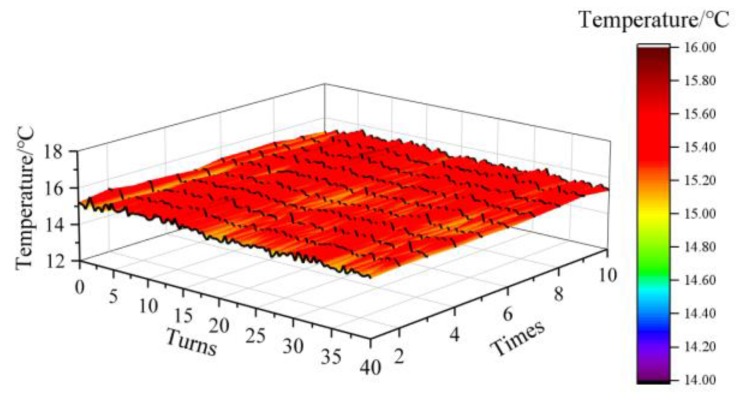
Winding original temperature.

Using the BOTDR to measure the BFS of the fiber, three sets of parallel tests are performed, and the BF curve is obtained as shown in Figure 9 and recorded as the initial group, that is, Δ*ν_Β0_* n Equation (5). The standard deviation of the three parallel measurements is 0.499 MHz, the composite strain is 14.76με the range is ± 1 MHz, and the composite strain is ± 29.59με. The frequency shift curve in Figure 10 fluctuates due to the prestress that is applied to the fiber during the winding process, and the prestress is unevenly distributed over the entire length of the winding.

The ROTDR test uses a distributed fiber optic temperature detection system produced by Weihai Beiyang Optoelectronic Info-Tech Co., Ltd. (Weihai, China) The parameters are set as shown in Table 3:

Under these parameter settings, the test time is 10 s.

The indoor temperature is 15.2 °C measured by a standard thermometer. The winding temperature distribution was measured by ROTDR. The results of ten parallel measurements are shown in Figure 10 below, with a standard deviation of 0.1808 °C and a measurement accuracy of ± 0.5 °C (maximum error between the accurate value and the measured value).

### 4.2. Winding Strain Detection

**Figure 11:**
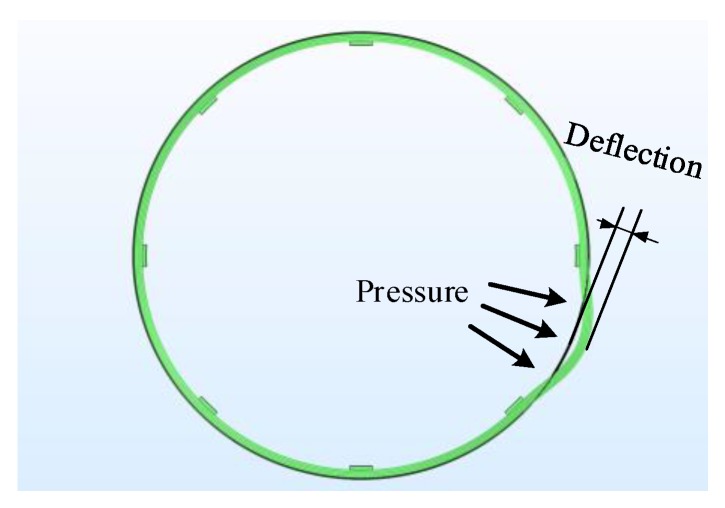
Schematic diagram of winding deformation.

**Figure 12:**
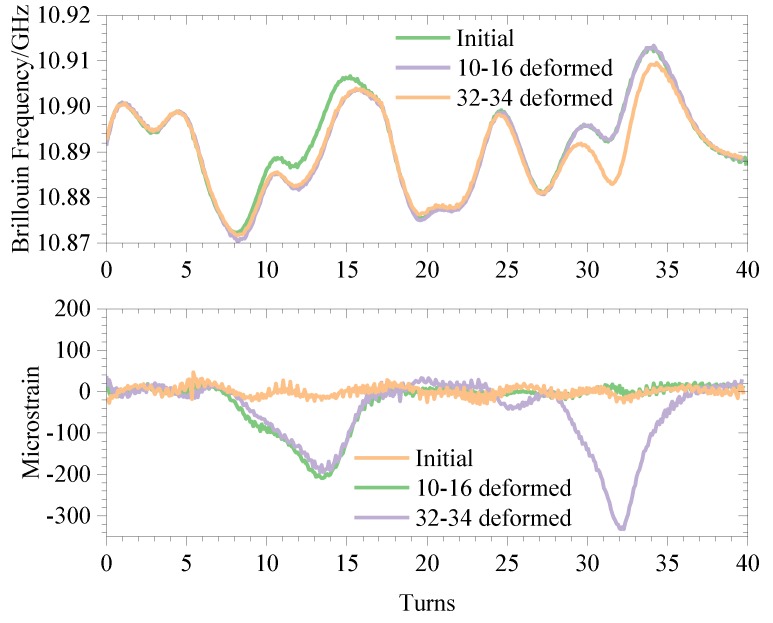
BOTDR detection curve.

Deformation experiments were carried out on the windings in the original state, and the convex strain was applied to the windings 10–16 turns and 32–34 turns, respectively, so that the corresponding diameter of the windings was increased by 10 mm and 17 mm, that is, the deflections are 10 mm and 17 mm, as shown in Figure 11. The Brillouin frequency shift curve was measured using BOTDR, as shown in Figure 12.

It can be seen from Figure 12 that when the winding is deformed, the Brillouin frequency shift curve will change, and the amount of change reflects the size of the deformation. As shown in Table 4, when the temperature is constant and the winding temperature is uniform, the BOTDR technology can be used alone to achieve the positioning and degree judgment of the winding deformation.

Since the experiments in this group are all at room temperature, the temperature change can be considered as 0, that is, Δ*Τ*=0 in Equation (5), so the Brillouin frequency shift is only related to strain.

### 4.3. Winding Temperature Test

According to Section 4.2, the BOTDR can accurately detect the deformation of the winding while the temperature remains constant, but the winding temperature distribution is not uniform during the actual operation of the transformer. We used the heating wire as the heat source to simulate the uneven distribution of the temperature of the winding. Combined with the two measurement methods of BOTDR and ROTDR, the temperature compensation of the Brillouin frequency shift and the distributed measurement of the temperature and strain of the winding have been explored.

The experimental steps are as follows:

(1) The winding wire of the 21st to 22nd windings is pushed out by about 15 mm, causing convex deformation. The BOTDR measurements were carried out under plastic deformation conditions, and the temperature distribution was measured by ROTDR to compensate for the Brillouin frequency shift caused by the uneven temperature. The obtained strain distribution curve was recorded as a deformation group.

(2) Adhere the two heating wires to the 4th−7th and 21st−24th turns of the winding and stick them to the outermost wire of the winding. The voltage is controlled to 20 V by the regulator to simulate the uneven distribution of the local overheating temperature of the winding. BOTDR and ROTDR measurements were performed after standing for 1 h to stabilize the temperature distribution. Using the ROTDR measured temperature data, the Brillouin frequency shift is compensated according to Equation (5), and the obtained strain distribution curve is recorded as a deformation and heating group.

(3) As a comparison, all of *T_i_* in Equation (5) are taken at room temperature, and temperature compensation is performed only at room temperature. A strain distribution curve that does not take into account the uneven distribution of the winding temperature is obtained, and is recorded as an uncompensated group.

**Figure 13:**
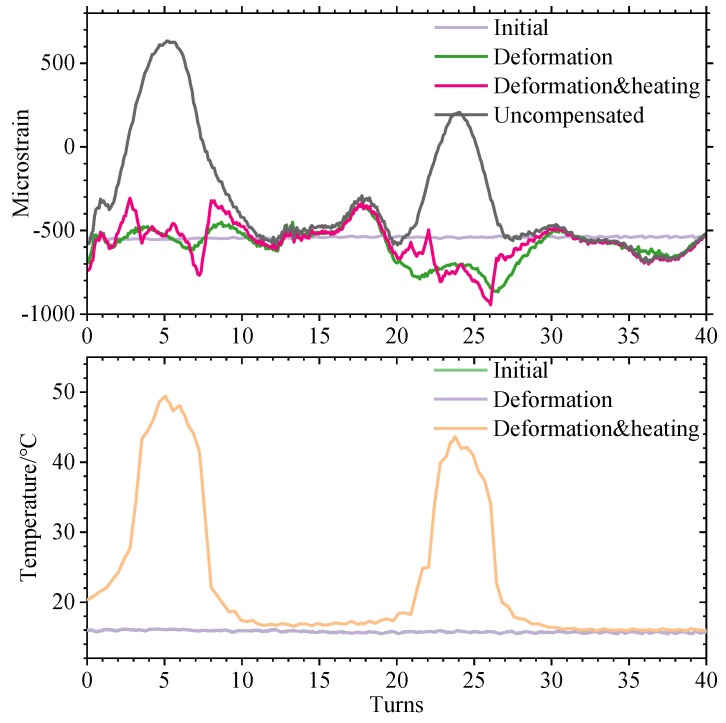
Schematic diagram of temperature compensation.

A comparison of the three sets of measurements with the initial curve is shown in Figure 13.

It can be seen from this test that simply using BOTDR technology to analyze the BFS to measure the winding deformation will cause misjudgment when the winding temperature distribution is uneven. If no temperature compensation measures are taken in this test, as shown in the uncompensated group, the temperature change at 3–7 turns of the winding will be incorrectly judged as deformation, causing unnecessary trouble to the maintenance work. During normal operation of the transformer, the winding temperature is generally gradually reduced from top to bottom, and the maximum temperature difference can reach 20 °C. If there is no compensation and the BFS caused by temperature is eliminated, the BOTDR technology will be unusable. When the temperature compensation is introduced, it can effectively compensate for the influence of uneven temperature distribution of the windings. As shown in the comparison between the deformation heating group and the deformation group in Figure 13, after removing the frequency shift caused by the temperature change, the measured winding strain distribution curve is basically the same as the winding strain distribution curve when the temperature is uniformly distributed. The influence of heating wire heating on the Brillouin frequency shift during the experiment is eliminated, and a more accurate deformation of the winding is obtained, as shown in Table 5.

### 4.4. Discussion and Error Analysis

The BOTDR equipment used in this experiment was set according to the parameters shown in Table 2. The measurement accuracy of the Brillouin frequency shift was about 1 MHz and the spatial resolution was 3 m, corresponding to about 1.4 turns on the winding. However, the winding deformation range measured by the BOTDR technology in this test is generally larger than the actual deformation area by 2–3 turns, which is equivalent to a length of about 5.5 m in the axial direction of the fiber. There are two main reasons for this reduction in spatial resolution:

(1) Brillouin scattering characteristics. It is generally considered that the Brillouin spectrum at a certain point is the superposition of the strain and temperature within a half optical pulse length [15]. Therefore, at the edge of the strain or temperature change, the range of strain detected by the BOTDR will be slightly larger than the actual.

(2) The winding is a whole; when a certain turn deformed, the surrounding turns will also be subjected to a certain tensile force to cause a certain deformation. The BFS of the fiber adhered to it will also change.

The error between the deformation group and the deformation and heating group in Figure 9, that is, the deviation of the temperature compensation is at most 200 με, which appears at the edge of the temperature change. The maximum and minimum values are +127 με and −98 με in the boxplot, respectively. There may be two reasons for this error:

(1) The smaller diameter of the heating wire may cause uneven temperature distribution of the winding, and there is a difference between the temperature of SMF and MMF, which causes an error in compensation.

(2) Due to the influence of the physical properties of Brillouin scattering, its response interval to a region with severe temperature changes is longer. The corresponding region of Raman scattering is relatively short, so over- and under-compensation will occur in the temperature change region.

## 5. Conclusions

1. Manufactured a wrapped winding model with built-in distributed sensing fiber, and used this wire to complete a helical 110 kV transformer winding model.

2. The solid mechanics simulation of the winding model verifies that the sensing fiber laid in the winding can realize the sensing of the winding strain, and the mean value of the stress on the fiber has a quadratic relationship with the degree of winding deformation, R^2^ = 0.9999. At the same time, it is verified that the temperature difference between the two sensing optical fibers is very small in the operation of the transformer, so the temperature compensation for the BFS can be realized.

3. Conducted a winding strain detection test under constant temperature conditions; the results show that accurate judgment of winding deformation can be achieved, and its positioning accuracy is within 5 m.

4. The winding was locally heated by the heating wire, and the strain was detected using the BOTDR-ROTDR combined measurement method. The strain detection accuracy was within 5 m, and the positioning accuracy of the center of deformation area on the winding was within 2 turns.

5. According to the research in this article, wrapped wire combined with BOTDR-ROTDR technology can realize the detection of winding deformation, and can eliminate the effect of temperature on strain detection. It provides a new technical route for future online monitoring of transformers.

## Figures and Tables

**Table 1:** Material Parameters for Simulation Model [13,26].

	Copper	ETFE	SiO_2_	Insulating Paper	Transformer Oil
Density(kg·m^−3^)	8940	2200	2203	780	860
Young’s Modulus (GPa)	126	0.4	73.1	0.3	-
Poisson’s Ratio(1)	0.34	0.4	0.17	0.35	-
Constant Pressure Heat Capacity (J·(kg·°C)^−1^)	385	1050	703	800	1870
Thermal Conductivity (W·(m·K)^−1^)	400	0.24	1.38	0.21	0.11

**Table 2:** BOTDR parameter settings.

Project	Parameter
**Pulse Width**	50 ns
**Sampling Resolution**	0.2 m
**Frequency Range**	10.75 GHz−11.05 GHz
**Sweep Step**	5 MHz
**Acquisition Length**	1 km
**Average Times**	2^13^
**Spatial Resolution**	3 m

**Table 3:** ROTDR parameter settings.

Project	Parameter
**Acquisition Length**	1 km
**Sampling Resolution**	0.8 m
**Spatial Resolution**	2 m

**Table 4:** Comparison of deformation test results.

Item		Unit/m	Unit/Turn
**1st deformation test**	Actual location	22.5–36	10–16
	Measuring position	18.8–36.8	9–16
**2nd deformation test**	Actual location	72.0–76.5	32–34
	Measuring position	63.8–82.0	30–36

**Table 5:** Comparison of deformation and heat-up test results.

Item		Unit/m	Unit/Turn
**Deformation test**	Actual location	47.25–49.5	21–22
	Measuring position	41.8–60.0	21–28
**Temperature test**	Actual location	(9.0–5.75) and (47.2–54.0)	(4–7) and (21–24)
	Measuring position	(5.8–17.0) and (44.4–56.7)	(4–8) and (23–26)
